# Substitutions in the β subunits of sickle-cell hemoglobin improve oxidative stability and increase the delay time of sickle-cell fiber formation

**DOI:** 10.1074/jbc.RA118.006452

**Published:** 2019-01-10

**Authors:** Fantao Meng, Tigist Kassa, Michael Brad Strader, Jayashree Soman, John S. Olson, Abdu I. Alayash

**Affiliations:** From the ‡ Laboratory of Biochemistry and Vascular Biology, Center for Biologics Evaluation and Research, Food and Drug Administration, Silver Spring, Maryland 20993 and; the § BioSciences Department, Rice University, Houston, Texas 77251

**Keywords:** hemoglobin, gene therapy, mass spectrometry (MS), hypoxia, oxidation-reduction (redox), redox reactions, sickle-cell disease

## Abstract

After reacting with hydrogen peroxide (H_2_O_2_), sickle-cell hemoglobin (HbS, βE6V) remains longer in a highly oxidizing ferryl form (HbFe^4+^=O) and induces irreversible oxidation of “hot-spot” amino acids, including βCys-93. To control the damaging ferryl heme, here we constructed three HbS variants. The first contained a redox-active Tyr in β subunits (F41Y), a substitution present in Hb Mequon; the second contained the Asp (K82D) found in the β cleft of Hb Providence; and the third had both of these β substitutions. Both the single Tyr-41 and Asp-82 constructs lowered the oxygen affinity of HbS but had little or no effects on autoxidation or heme loss kinetics. In the presence of H_2_O_2_, both rHbS βF41Y and βF41Y/K82D enhanced ferryl Hb reduction by providing a pathway for electrons to reduce the heme via the Tyr-41 side chain. MS analysis of βCys-93 revealed moderate inhibition of thiol oxidation in the HbS single F41Y variant and dramatic 3- to 8-fold inhibition of cysteic acid formation in rHbS βK82D and βF41Y/K82D, respectively. Under hypoxia, βK82D and βF41Y/K82D HbS substitutions increased the delay time by ∼250 and 600 s before the onset of polymerization compared with the rHbS control and rHbS βF41Y, respectively. Moreover, at 60 °C, rHbS βK82D exhibited superior structural stability. Asp-82 also enhanced the function of Tyr as a redox-active amino acid in the rHbS βF41Y/K82D variant. We conclude that the βK82D and βF41Y substitutions add significant resistance to oxidative stress and anti-sickling properties to HbS and therefore could be potential genome-editing targets.

## Introduction

Sickle-cell disease (SCD)^3^ is a pleiotropic disorder marked by hemolytic anemia and vascular-occlusive complications (1, 2). This genetic condition is caused by a single point mutation at the β6 position of Hb in which Glu is replaced by Val. Under conditions of low-oxygen tension, deoxy-HbS molecules form long fibers leading to the characteristic sickle shape of red cells. Sickling and unsickling cycles (as RBCs pass through narrow capillaries) lead some cells to rupture and release Hb and intracellular components into plasma. Vaso-occlusion typically occurs after endothelial activation, expression of adhesion molecules, and subsequent adhesion of leukocytes (and sickled RBCs) to the vascular wall (3). Although polymerization of intraerythrocytic deoxy-HbS is the primary molecular event that leads to hemolytic anemia, evidence is emerging that oxidation side reactions of Hb within SS RBCs, RBC-derived microparticles (MPs), and the released free Hb contribute to the complex pathophysiology of the disease (4).

The unique oxidative environment within these SS RBCs acts as an incubator for various reactive redox forms of Hb. Because of the catalytic nature of these reactions, Hb is proposed to participate (inside and outside RBCs) in pseudoenzymatic radical side reactions (5). In sickle-cell blood, both intraerythrocytic and free HbS oxidize at higher rates than normal HbA and undergo oxidative changes that include the formation of a persistent and highly oxidizing ferryl state (HbFe^4+^=O) as part of this pseudoperoxidative cycle. In addition to targeting its own β subunits, ferryl Hb and its associated radicals (^•^HbFe^4+^=O) actively interact with other biological molecules in membranes and organelles, including the mitochondria (6). The oxidative milieu in both RBCs and MPs provides a fertile ground for acceleration of oxidative degradation of Hb. Reactive oxygen species (ROS) fuel the Hb pseudoperoxidase redox cycle (Fe^2+^ → Fe^4+^ → Fe^3+^), which can be a source of ROS (5, 7, 8).

We have recently reported a mechanistic link between Hb-dependent oxidative reactions and erythrocyte membrane changes (particularly in the band 3 proteins). Additionally, we showed that events within MPs mimic that of the original red cells, including post-translational modifications such as ubiquitination of lysines (βLys-96 and βLys-145) and irreversible oxidation of Cys-93 in β subunits of HbS (9).

The ability of some Hb variants (*e.g.* fetal and Ibadan Hbs) to impair HbS polymerization by diluting out HbS is well documented (10). Moreover, naturally occurring human Hb variants often adapted to either resist oxidative damage (which leads to the patient being asymptomatic) or in some cases develop into a full circulatory disorder (11). For example, the Hb Providence mutation (βK82D) was observed as a second mutation in a person with the βE6V trait. This patient showed no evidence of sickle-cell disease (SCD), and the Hb of this patient migrated similarly to HbA in electrophoresis experiments (12). One purpose of this work was also to try to understand at the molecular level why the βK82D mutation reduces considerably the SCD symptoms in this patient (12).

Our group has previously shown that HbProv (βK82D) is more resistant to damage by H_2_O_2_ than native HbA because of its ability to better quench the ferryl radicals through an efficient ferric/ferryl pseudoperoxidase cycle (13). We subsequently engineered this unusual oxidative stability into a genetically cross-linked human tetramer as a potential oxygen therapeutic (14). The HbProv mutation conferred more resistance to degradation by H_2_O_2_ by inhibiting oxidation of the β93 cysteine side chain. To test whether this property could mitigate sickling and degradation of HbS, we constructed an rHb containing the double mutant, βE6V/K82D.

**Figure 1.**
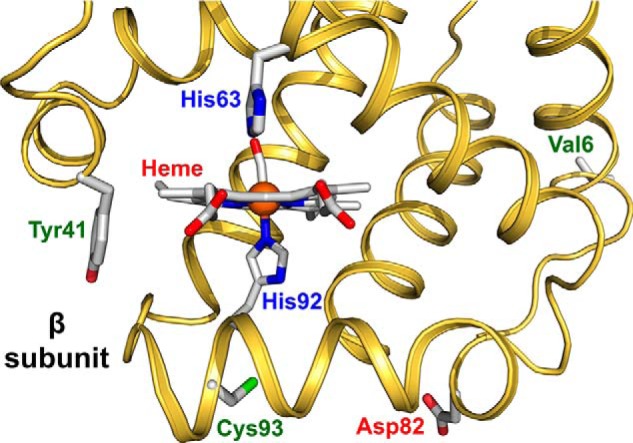
**Model structure of HbS showing the sites of mutations in the β subunit.** The *ribbon* drawing was generated from the coordinates for the crystal structure of the CO form of HbS (PDB code 5E6E). The side-chain orientations for Val-6, His-63, His-92, and Cys-93 were taken from this structure, and bound CO is shown attached to the iron atom (*orange sphere*). The side-chain orientation of Tyr-41 was generated by adding the OH group to Phe-41 in the original structure. The orientation of the Asp-82 was taken from the structure of rHb0.1Prov-CO (PDB code 5SW7). Distance from the heme to Tyr-41 is 10.4 Å, whereas Asp-82 is located 18.1 Å away from the heme. The structure was drawn using the PyMOL Molecular Graphics System, Version 2.0 (Schrodinger, LLC).

Redox-active tyrosine residues can facilitate electron transfer between endogenous antioxidants and oxidative ferryl heme species (15–17). A suitable residue is present in the Hb α subunit (Tyr-42) but absent from the homologous position in the β subunit (Phe-41). The naturally occurring Hb variant Mequon, βF41Y (Fig. 1), contains the active tyrosyl side chain in both subunits. The single βF41Y substitution has also been constructed in rHb and shown to reduce heme iron–mediated oxidative reactivity (18).

In this report, we engineered these two mutations into recombinant HbA, creating two double mutants and one triple mutant, *i.e.* βE6V/F41Y, βE6V/K82D, and βE6V/F41Y/K82D, all three of which carry the HbS mutation (Fig. 1). Our data show that substitution of Phe at position 41 with a redox-active Tyr in the β subunit provides an effective electron pathway to reduce the levels of ferryl heme, particularly in the presence of ascorbate as a physiologically relevant, mild reducing agent. Substitution of Asp for Lys at the β82 position of HbS provided resistance to polymerization and denaturation as measured by significant increases in gelation time during hypoxia and enhanced thermal stability of the protein. The combined benefits of the two mutations in HbS potentially serve as a useful model to further investigate the role of these two replacements in ameliorating sickling and improving oxidative stability in the protein and could also provide strategies for potential drug development and gene therapy interventions, if reversal of the βV6E mutation is difficult.

## Results

### Electrophoretic mobilities and chromatography confirm site-specific mutations in sickle-cell hemoglobin constructs

Isoelectric focusing (IEF) analysis confirmed amino acid substitutions in these mutant proteins. An isoelectric point (pI) of ∼7.1 for rHbS (E6V) was estimated, which is close to that of native HbS and the βF41Y mutation. The introduction of the Providence mutation, βK82D, into HbS with an additional negative charge brought the pI closer to that of HbA (pI ≈6.9) as described previously by Gale *et al.* (12). No changes were seen in the triple mutant (rHbS βF41Y/K82D), which may reflect the dominance of Asp over the Tyr mutation (data not shown). We used reversed-phase HPLC analyses (6) to monitor subunit integrity in these mutant proteins. No major changes were noted in the peaks of their α and β subunits. The heme peak of all samples remained constant and served as an internal control for monitoring the effects of the mutations at the time of elution (data not shown). The subunit mutations for all recombinant proteins were also verified by MS (see under “Experimental procedures”).

### Oxygen-binding curves of hemoglobin S mutants are right-shifted

**Figure 2.**
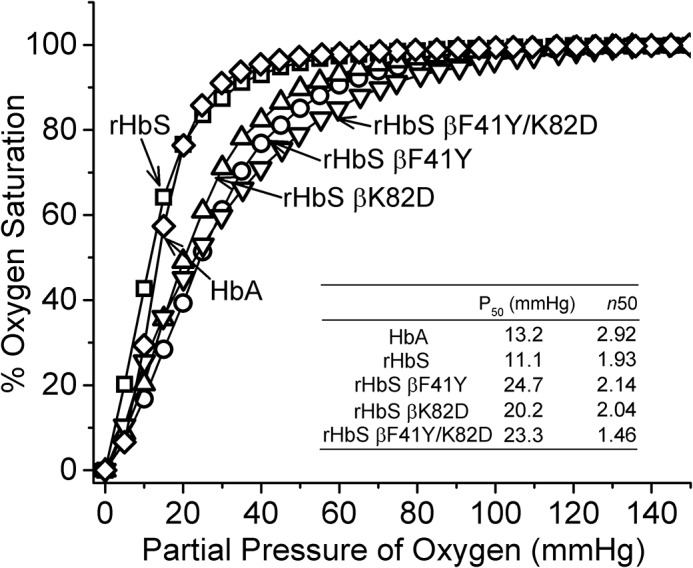
**OECs for mutant HbS.** The *inset table* summarizes the *P*_50_ and Hill coefficient (*n*_50_) of the HbS variants. The OECs were measured in a Hemox analyzer. The experiments were carried out with 70 μm (heme) Hb in 10 mm phosphate buffer, pH 7.4, containing 0.1 m NaCl, anti-foaming agent, and the Hayashi enzymatic reducing system (d-glucose 6-phosphate, glucose-6-phosphate dehydrogenase, and β-NADPH monosodium salt, ferredoxin, ferredoxin-NADP^+^ reductase, and catalase (bovine)) at 37 °C.

Oxygen equilibrium curves (OECs) for all recombinant proteins in this report were sigmoidal, and cooperativity (*n*_50_) was largely maintained in these proteins (Fig. 2). The OEC curve for rHbS was similar to that of native HbS and HbA. However, both the βF41Y and βK82D mutations caused right-shifts, with increases in calculated *P*_50_ values compared with the rHbS control (19, 20). The oxygen-affinity value for rHbS βF41Y/K82D is close to that reported earlier by Dumoulin *et al.* (21) for the same substitutions in an HbA background (see *inset* of Fig. 2, *last line*).

### Autoxidation reactions of hemoglobin S mutants reveal similar kinetics to the WT protein

**Figure 3.**
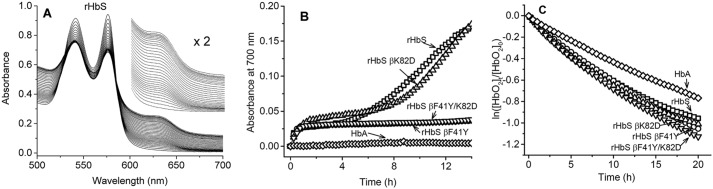
**Autoxidation kinetics and early oxidative changes in hemoglobin S mutants.** *A,* spectra collected during autoxidation reaction of rHbS in PBS at 37 °C. *B,* absorbance changes at 700 nm during the autoxidation of HbA and the HbS mutants, which reflect onset of precipitation. *C,* first-order plots for the autoxidation reactions of rHbS mutants in PBS at 37 °C.

Under air-saturated conditions, ferrous oxy-HbS, like oxy-HbA, spontaneously oxidizes to ferric (metHb). Fig. 3*A* shows typical kinetic spectral scans of the changes captured when 60 μm oxy-rHbS undergoes oxidation during incubation in PBS at 37 °C for 24 h (6).

In these experiments, there was a steady decline in the peaks at 541 and 576 nm and a corresponding increase in the 630-nm peak, both of which are indicative of the formation of metHb. For HbA, there was little or no change in the isosbestic points suggesting a simple transition from oxy-Hb to metHb with no intermediates. However, in HbS and rHbS solutions there was a steady increase in the baseline (λ = 700 nm), due to the polymerization and precipitation of the protein toward the end of the incubation period (24 h). Fig. 3*B* captures the increase in light scattering at 700 nm due to polymerization/precipitation in the rHbS solutions. It is interesting to note that both rHbS βF41Y and βF41Y/K82D were much more stable than either rHbS or rHbS βK82D.

Time courses for the spontaneous oxidation of HbS variants were measured for over 24 h, and the decay of the oxy forms of all the Hbs with time is shown in Fig. 3*C*. Individual autoxidation rate constant estimates were obtained by fitting the time courses to a single exponential expression. The autoxidation rate for rHbS was calculated to be 0.069 h^−1^, which is nearly identical to that reported previously by our group for native HbA at the same heme concentrations (6). The rates of autoxidation of rHbS βK82D and βF41Y/K82D were somewhat greater (≤2-fold) than those for native HbA and HbS (Table 1).

**Table 1:** **Autoxidation, chemical oxidation, and heme loss kinetics for hemoglobin S mutants**

	Autoxidation rate	Heme loss rate		*k*_oxid_*^a^*	[sulfHb]*^b^* at 30 min	Ferryl reduction*^c^*		
		*k*_1_	*k*_2_			Autoreduction, *k*_auto_	50 μm ascorbate, *k*_1_	50 μm ascorbate, *k*_2_
	*h*^−*1*^	*h*^−*1*^		*min*^−*1*^	μ*m*	*min*^−*1*^		
HbA	0.040 ± 0.002	9.0 ± 1.3	1.0 ± 0.1	0.13 ± 0.01	14.3 ± 0.3	0.052 ± 0.001*^d^*	0.359 ± 0.007	0.032 ± 0.001
Native HbS	0.047 ± 0.002	15.5 ± 1.9	1.7 ± 0.3	0.14 ± 0.01	14.1 ± 0.2	0.048 ± 0.001	0.326 ± 0.006	0.022 ± 0.001
rHbS	0.069 ± 0.003	12.4 ± 1.1	2.1 ± 0.1	0.18 ± 0.01	16.1 ± 0.5	0.046 ± 0.001	0.319 ± 0.023	0.021 ± 0.000
rHbS βF41Y	0.067 ± 0.002	14.0 ± 1.3	3.3 ± 0.2	0.19 ± 0.01	9.5 ± 0.2	0.058 ± 0.001	0.426 ± 0.033	0.045 ± 0.001
rHbS βK82D	0.075 ± 0.006	16.1 ± 0.8	2.5 ± 0.2	0.24 ± 0.01	16.2 ± 0.4	0.051 ± 0.001	0.311 ± 0.028	0.014 ± 0.001
rHbS βF41Y/K82D	0.080 ± 0.004	9.8 ± 0.5	3.8 ± 0.4	0.24 ± 0.01	9.2 ± 0.3	0.057 ± 0.001	0.455 ± 0.043	0.032 ± 0.001

*^a^* Data were calculated from Fig. 5, 60 μm Hb + 60 μm H_2_O_2_ with a spectrophotometer.

*^b^* Data were calculated from Fig. 6*B*.

*^c^* Data were calculated from Fig. 7. 5 μm ferryl Hb reacted with 0 or 50 μm ascorbate in PBS at room temperature.

*^d^* Initial autoreduction rate is shown.

### Ferric forms of HbS variants show similar rates of hemin loss

**Figure 4.**
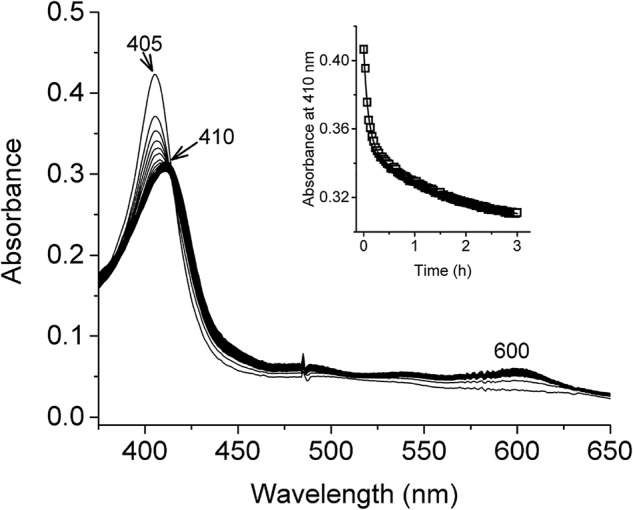
**Heme transfer from met-rHbS to apoH64Y/V68F myoglobin mutant.** Spectral changes observed during the reaction of met-rHbS with apoH64Y/V68F for 16 h at 37 °C. Spectra was recorded every 2 min. *Inset* shows time course of the reaction; absorbance changes at 410 nm were fitted to a double-exponential equation to calculate the rate of heme release from rHbS and its mutants.

To assess the rate of hemin loss, we monitored hemin release from these proteins using the high-affinity H64Y/V68F apoMb hemin-scavenging reagent (22). Fig. 4 shows an example of spectral changes recorded as hemin is transferred from an rHbS variant to the apomyoglobin double mutant at 37 °C over a period of 16 h. The *inset* in Fig. 4 shows the time course of the absorbance changes at 410 nm for rHbS. The time courses were normalized and fitted to a double-exponential decay, where *k*_1_ and *k*_2_ represent the rate constants (h^−1^) for the fast (β chain) and slow (α chain) phases, respectively (22). The values for the hemin loss rate constants are reported in Table 1, and as in the case of the autoxidation, all the HbS variants showed similar β (10–16 h^−1^) and α (2–3 h^−1^) hemin dissociation rate constants, respectively.

### Tyrosine 41 as a redox-active center reduces ferryl heme and in combination with aspartate 82 provides oxidative stability in the β subunits of hemoglobin S

**Figure 5.**
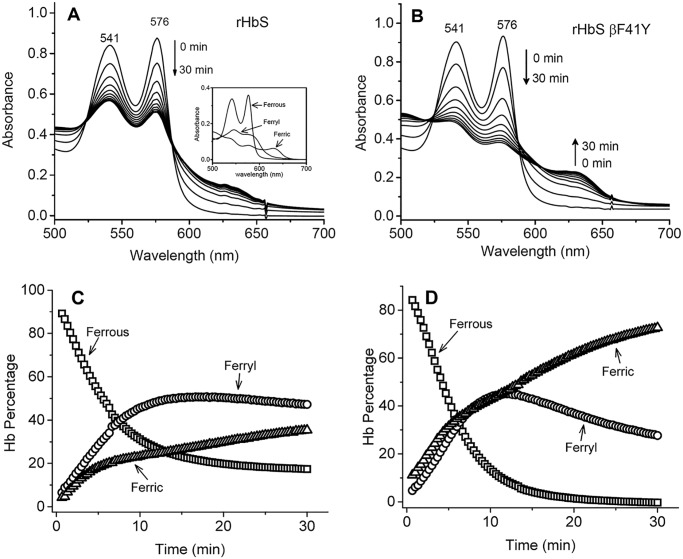
**Oxidation of hemoglobin S and its mutants by hydrogen peroxide.** *A* and *B,* spectral changes during the oxidation of rHbS and its Tyr-41 mutant by peroxide over a 30-min interval. The *inset* in *A* represents the deconvoluted spectra for the three hemoglobin components at the first 10-min reaction time point. *C* and *D,* fractional changes of ferrous, ferric, and ferryl in rHbS and its Tyr-41 mutant during the incubation of 60 μm Hb with 60 μm H_2_O_2_ in PBS at room temperature.

H_2_O_2_ reactions with the oxy form of rHbS mutants were carried out in a rapid-scanning spectrophotometer. We used increasing H_2_O_2_ ratios over heme equivalents (*i.e.* 1, 2.5, 5, and 10 H_2_O_2_/heme). During a typical experiment for rHbS and rHbS βF41Y (Fig. 5, *A* and *B*), the initial oxidation of ferrous heme was seen by a gradual loss of the α and β visible absorbance bands at 576 and 541 nm, the appearance of a ferryl intermediate with absorbance peaks at 545 and 580 nm, and a flatter region between 600 and 700 nm. The ferryl peak then reverts back over 30–60 min to metHb with peaks at 541, 576, and 630 nm, completing a pseudoperoxidase cycle. Fractional changes of oxy, met, and ferryl Hbs (Fig. 5, *C* and *D*) were calculated from multicomponent analysis of the observed spectra for all mutant samples at different ratios of heme using standard extinction coefficients for each redox species (see *inset* in Fig. 5*A*) (23).

The oxidative pathway represented by the data in Fig. 5 follows the well-tested model that we previously reported for human Hb and mammalian Mbs (24, 25). There are three distinct steps: 1) initial oxidation of oxy to ferryl Hb; 2) autoreduction of ferryl intermediate to ferric Hb; and 3) with excess H_2_O_2_, the reaction of metHb with additional H_2_O_2_ results in a pseudoperoxidase cycle (24). In the first step, ferrous rHbS is rapidly converted to a ferryl intermediate after addition of H_2_O_2_, with an apparent rate, *k*_oxid_, on the order of 0.1 to 0.2 min^−1^ for all the HbS variants examined. As shown in Table 1, the *k*_oxid_ for native and recombinant HbS is measurably smaller (30–50%) than that for the HbS single and double mutants. The higher rates for the variants are readily explained by their higher *P*_50_ values as we described earlier (24). The first step in the reaction of H_2_O_2_ with HbO_2_ requires dissociation of bound O_2_, and thus the rate of this reaction is roughly proportional to the *P*_50_ of the Hb sample at low H_2_O_2_ concentrations.

**Figure 6.**
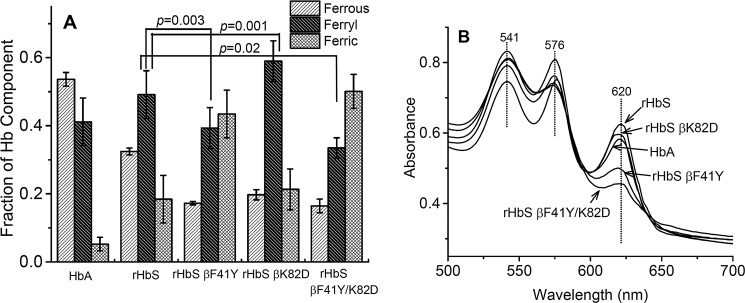
**Reactions of sickle-cell hemoglobin and its mutants with peroxide and sodium sulfide.** *A,* relationship between the different redox states (ferrous, ferric and ferryl) of sickle hemoglobin mutants during the 30 min of oxidation with peroxide (Hb/H_2_O_2_ = 60:60 μm). The fractions of each species were calculated by integrating the areas under the curves (for each Hb species) in Fig. 5, *C* and *D. B,* spectral changes in the visible region after oxidation of sickle-cell hemoglobin and its mutants with peroxide (Hb/H_2_O_2_ = 60:60 μm) and subsequent derivatization with sodium sulfide (Na_2_S) (2 mm).

Fig. 6*A* compares the relative fractions of the redox species 30 min after the addition of the 1:1 ratio of H_2_O_2_/heme. For the four proteins examined, the remaining ferrous heme was lower for the rHbS mutants due to their 20–30% faster initial oxidation rates. The rHbS βF41Y and βF41Y/K82D had less ferryl intermediate than rHbS, whereas the βK82D mutant contained the highest amount of ferryl intermediate during the 30-min incubation time, despite the fact that the autoreduction rates (*k*_auto_) for the ferryl to ferric forms of all mutants were in the range of 0.05 to 0.06 min^−1^ (Table 1).

Ferryl heme iron can be derivatized with Na_2_S to form a stable sulfHb form that can be monitored and quantified spectrophotometrically based on its strong 620-nm absorption band. Fig. 6*B* shows again that the rHbS βF41Y and βF41Y/K82D mutants contained 40–50% less sulfheme (∼9–10 μm) when the reaction was quenched, and Na_2_S was added at the 30-min time point. The highest sulfHb concentrations were measured for native and recombinant HbS and rHbS βK82D (14–16 μm).

Tyr residues can act as redox mediators by one-electron cycling between ferryl and ferric forms in the presence of exogenous electron donors. This electron transfer pathway by Tyr-42 in α subunits has been shown to enhance the rate of ferryl reduction, and a kinetic model based on monitoring spectral changes following ascorbate addition has been reported by Reeder *et al.* (15–17). The observed rate of reduction showed a two-phase hyperbolic dependence on [ascorbate], likely due to the presence of high- and low-affinity sites for transient binding of the reducing agent (26).

**Figure 7.**
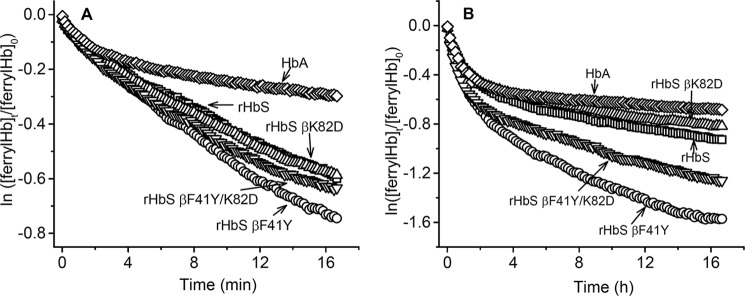
**Kinetics of reduction of ferryl heme in solutions of sickle-cell hemoglobin mutants by ascorbate.** *A,* autoreduction of ferryl hemoglobin in PBS at room temperature as monitored in the stopped flow. *B,* reduction of ferryl heme (5 μm) after mixing with 50 μm ascorbate in PBS at room temperature and monitoring spectral changes with a stopped-flow spectrophotometer.

Fig. 7 compares time courses for auto- and ascorbate-dependent reduction of the ferryl states of the rHbS mutants. As shown in Fig. 7*A*, the initial rates of auto-reduction are the same for all the HbS variants, and the reactions of these mutants appear monophasic compared with that for HbA. Reduction of ferryl Hb by ascorbate exhibits a biphasic time course (*k*_1_ = 0.052 min^−1^ and *k*_2_ 0.008 min^−1^) (Fig. 7*A*). In contrast to autoreduction of the HbS variants, the time courses for ascorbate reduction vary significantly among the mutants, and all show two distinct phases (Fig. 7*B*), which were fitted to a two-exponential expression.

Ascorbate reduction rates for the first and second phases are listed for all four mutants in Table 1. The biphasic nature of these plots appears to reflect fast reduction of the ferryl heme in α subunits and slower reduction in β subunits by ascorbate (17). The data in Table 1 show that the βF41Y mutation increases the rate of ascorbate reduction in HbS β subunits over 2-fold as was seen in HbA β subunits. This βF41Y replacement also enhances ascorbate reduction 3-fold in rHbS K82D β subunits (*k*_2_ values in Table 1, rows 3 and 4).

### βAsp-82 increases the delay time prior to fiber formation in hemoglobin S mutants

**Figure 8.**
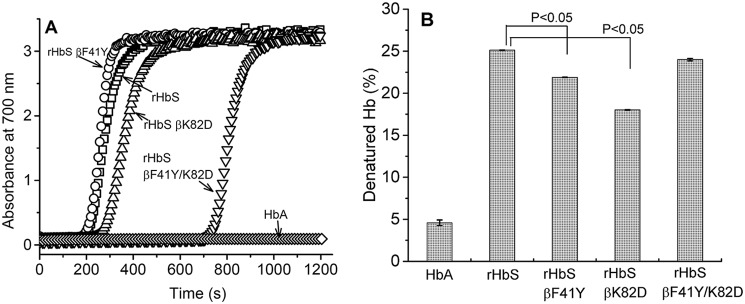
**Kinetics of polymerization and heat stability of hemoglobin A, hemoglobin S, and its mutants.** *A,* kinetics of deoxy-Hb polymer formation. Solutions of deoxy-Hb (100 μm) in 1.8 m phosphate buffer were rapidly heated from 0 to 30 °C, and polymer formation was assessed by light scattering at 700 nm. *B,* various mutants were heated at 60 °C for 10 min in 100 mm phosphate, pH 7.4, and the percent of precipitated hemoglobin was calculated after centrifugation. Turbidity was also measured spectrophotometrically by recording the absorbance at 700 nm of the concentrated hemoglobin solutions (2 mm).

Anaerobic temperature-jump experiments were carried out to establish the polymerization kinetics of rHbS and its mutant derivatives using the well-established method introduced by Adachi *et al.* (27). The increase in light scattering (turbidity) at 700 nm was plotted as a function of time to generate typical sigmoidal curves, as shown in Fig. 8*A*. After a short lag time, there was an elevation in absorbance at this wavelength indicating the beginning of aggregation and precipitation. Both rHbS and rHbS βF41Y formed aggregates after ∼200 s upon addition of dithionite to induce hypoxic conditions. rHbS βK82D aggregated after ∼257 s. Aggregation of rHbS βF41Y/K82D occurred after a much longer lag phase of 700 s suggesting a significant inhibition of polymerization by the double mutations.

### βK82D replacement increases thermal stability of HbS

Thermal and mechanical instabilities are some of the biophysical hallmarks that differentiate HbS from normal HbA (28). We therefore examined the thermal stability of the oxy forms of the HbS mutants in a PCR thermal cycler, which is known to have a better temperature control than conventional spectrophotometers (29). After incubating for 10 min at 60 °C, 25.12 ± 0.02% of rHbS was denatured, which is close to the reported values for native HbS and much higher than values obtained for HbA (4.8%). The extent of denaturation for the rHbS variants under these conditions was as follows: rHbS βK82D = 18.02 ± 0.03%, rHbS βF41Y = 21.89 ± 0.03%, and rHbS βF41Y/K82D = 24.0 ± 0.14% of denatured protein (Fig. 8*B*). Compared with the other mutants, the single rHbS βK82D mutant was shown to have a relatively lower extent of denaturation, suggesting better thermal stability (Fig. 8*B*).

### LC/MS/MS analysis reveals a reduction in irreversible oxidation of βCys-93 in the Tyr-41 and Asp-82 mutants

**Figure 9.**
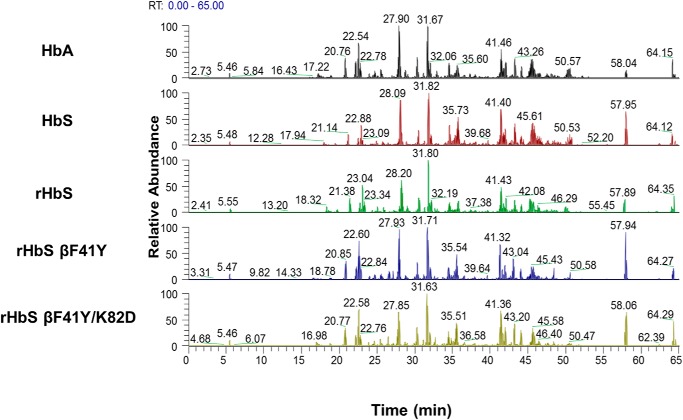
**Typical subset of tryptic digestion profiles (base peak chromatograms) for human hemoglobins S and A (controls) and the hemoglobin S mutant (E6V/F41Y) after peroxide treatment.** Base peak chromatograms listed for HbA, native HbS, rHbS, rHbS βF41Y, and rHbS βF41Y/K82D after parallel experimental treatment with 2.5× H_2_O_2_. Each chromatogram shows similar elution peaks detected during MS analysis. The similarity in peaks and retention times indicates and validates trypsin digestion efficiency for each run.

LC/MS/MS analysis was utilized to confirm that all recombinant mutant Hbs used in this study were catalase-free and that each sample had similar trypsin digestion efficiency. Fig. 9 illustrates a typical subset of base peak chromatograms (each chromatogram represents a separate data acquisition) identified for tryptic peptides eluting off a C18 reverse-phase column coupled on-line to the mass spectrometer. These tryptic digestion profile comparisons (performed under parallel experimental conditions) illustrate similarity in detected peaks and retention times indicating that the trypsin digestion efficiency was equivalent between each run. After confirming optimal trypsin digestion efficiency, quantitative MS was used to evaluate the impact of H_2_O_2_-induced oxidation by comparing oxidation of hot-spot amino acids in all four recombinant HbS constructs. LC/MS/MS analysis was utilized to target the hot spot containing charge states representing peptides that were reproducibly identified by Mascot database searches (Fig. 9). The amino acid residues identified in this study correlated well with previously published data (6, 14, 30) and include the following: βCys-93, βMet-55, βCys-112, βTyr-145, αCys-104, αTyr-42, and βTyr-35. We focused our analysis to include cysteine residues of interest, including βCys-93, βCys-112, and αCys-104 (see Tables 2 and 3).

**Table 2:** **All hot spot peptides and cleavage variants analyzed in this study (including different charge states)** The peptide representing residues 65–104 contains Asp-82 and Cys-93.

Peptides	Modified residue	(+) Charge state	*m*/*z*
^83^GTFATLSELHCDK^95^	βCys-93	2	735.33
^83^GTFATLSELHCDKLHVDPENFR^104^	βCys-93	3	860.06
		4	645.31
		5	518.25
^105^LLGNVLVCVLAHHFGK^120^	βCys-112	3	589.99
		2	884.48
	βCys-93	3	1036.83
^65^KVLGAFSDGLAHLDNLDGTFATLSELHCDKLHVDPENFR^103^		4	777.87
		
^100^LLSHCLLVTLAAHLPAEFTPAVHASLDK^127^	αCys-104	4	754.66

**Table 3:** **Hydrogen peroxide-mediated oxidation of the cysteine groups in hemoglobin S mutants**

	βCys-93 oxidation		βCys-112 oxidation		αCys-104 oxidation	
	2.5× H_2_O_2_	10× H_2_O_2_	2.5× H_2_O_2_	10× H_2_O_2_	2.5× H_2_O_2_	10× H_2_O_2_
HbA	22.1 ± 1.3%	51.1 ± 2.5%	2.9 ± 0.8%	19.5 ± 1.5%	1.6 ± 0.4%	7.9 ± 1.4%
Native HbS	34.9 ± 2.2%	62.4 ± 2.9%	17.6 ± 1.4%	25.2 ± 2.3%	5.4 ± 1.1%	11.7 ± 0.6%
rHbS	37.4 ± 1.5%	61.7 ± 0.7%	18.4 ± 1.0%	27.2 ± 2.1%	6.6 ± 0.9%	12.8 ± 0.8%
rHbS βF41Y	33.7 ± 3.5%	57.3 ± 1.7%	19.6 ± 1.9%	23.0 ± 2.3%	4.8 ± 0.8%	12.0 ± 1.3%
rHbS βK82D	11.9 ± 2.0%	22.1 ± 1.3%	20.8 ± 1.5%	23.4 ± 0.4%	6.2 ± 0.8%	10.7 ± 0.8%
rHbS βF41Y/K82D	4.6 ± 0.6%	9.1 ± 1.1%	19.7 ± 0.7%	25.4 ± 3.3%	6.6 ± 2.2%	11.4 ± 0.4%

**Figure 10.**
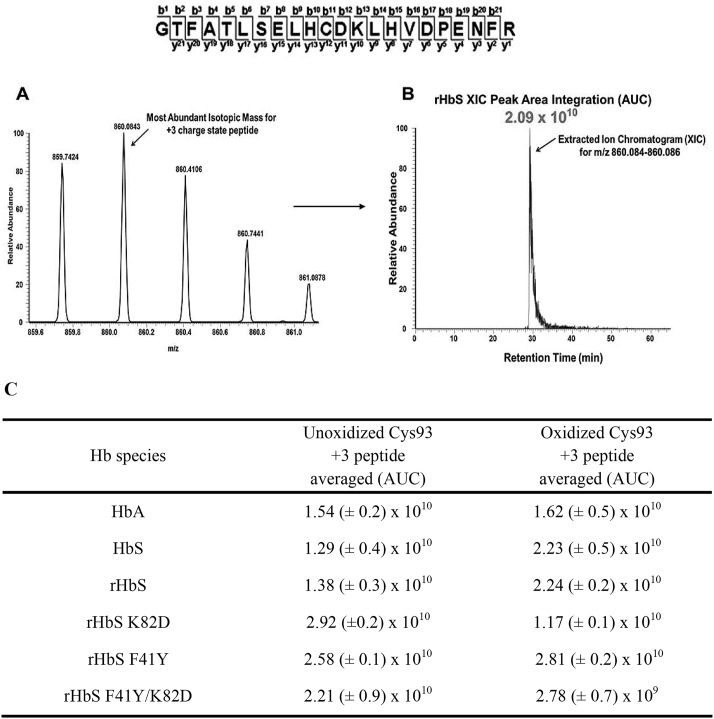
**Representative XIC data of the oxidized Cys-93 tryptic peptide rHbS (residues 83–104) after experimental treatment with 10× H_2_O_2_.** For quantitative MS experiments, the charge state isotopic profile of all hot-spot peptides (and their different charge states listed in Table 2) was analyzed as shown in this figure to quantify changes under different oxidative conditions. The panels in this figure represent data after experimental treatment with 10× H_2_O_2_. *A,* isotopic profile for the Cys-93 containing +3 charge state peptide (860.08 *m*/*z*) listed above (residues 83–104). *B,* representative XIC and peak area integration for the oxidized rHbS Cys-93 peptide (residues 83–104) generated from the ion current of the most abundant monoisotopic peak shown in *A. C,* averaged XIC peak area integration values (intensity values from three technical replicates) for the +3 charge state unoxidized and oxidized Cys-93–containing peptide (residues 83–104) for HbA, HbS, rHbS, rHbS K82D (residues 65–104), rHbS F41Y, and rHbS F41Y/K82D (residues 65–104), respectively.

Extracted ion chromatograms (XICs) were generated from the most abundant monoisotopic peak of each peptide isotopic profile, and the resulting ratio differences were compared. For example, the most abundant mono-isotopic peak (860.084 *m*/*z*) represented in Fig. 10*A* for the +3 βCys-93 peptide, GTFATLSELHCDKLHVDPEN-FR, was used to construct the resulting XIC (Fig. 10*B*). Because βCys-93 exists in either the oxidized or unoxidized form, the relative abundance of both isoforms was calculated based on the sum of the XIC peak area from all charged isoforms of βCys-93 peptides. As shown in Fig. 10*C*, XIC peak area integration values for βCys-93 peptides allowed quantification of oxidized βCys-93. XICs were generated in a similar manner for all hot-spot peptide charge states listed in Table 2. Because the data in Fig. 10*C* represent only the +3 charge state of the unoxidized and oxidized βCys-93 peptide, the averaged integration values reflect the overall trend associated with oxidative changes seen in Table 3. However, the calculated ratio values observed in Table 3 are based on the sum of XIC numerically integrated peak area of all βCys-93 peptide charge states (oxidized and unmodified) to be 100%.

As shown in Table 3, H_2_O_2_ addition (2.5- and 10-fold in excess) led to increased oxidation of all three hot-spot residues. Despite increased oxidation for all three residues, only βCys-93 oxidation was differentially affected by the HbS mutations. The βK82D substitution decreased βCys-93 oxidation in HbS significantly, resulting in a 3-fold reduction in cysteic acid formation. This result is similar to what we have recently observed when studying the influence of the Providence mutation (βK82D) by itself on hot-spot oxidation in genetically cross-linked Hb (rHb0.1) (14). The βF41Y mutation alone did not have much impact on βCys-93 oxidation in HbS, which is more reactive than HbA. However, the double mutant rHbS βF41Y/K82D showed a remarkable 7-fold decrease in βCys-93 oxidation, indicating that the presence of both F41Y and K82D had a much greater than additive effect. These results suggest strongly that both mutations reduce βCys-93 oxidation and/or reactivity through some subtle structural changes and perhaps reduction of the ferryl species via the presence of the βTyr-41 side chain.

## Discussion

There is growing recognition that SS RBCs experience oxidative stress throughout their lifetimes (31) and, unlike their normal AA counterparts, retain some mitochondria after maturation, which can be a major source for ROS (32). Consequently, the oxidative milieu, which also continues inside the circulating RBC-derived MPs, provides a fertile ground for the acceleration of HbS oxidation reactions (9). Hb-dependent oxidation reactions, including the formation of the highly-reactive ferryl heme within SS RBCs, have been shown to drive RBC membrane alterations, including Hb-mediated band 3 interactions and subsequent MP formation in transgenic SCD mice (9).

SCD is characterized by chronic hemolysis, which results in the accumulation of Hb and free heme released from lysed RBCs and MPs in circulation (33). Some of the more recent intervention strategies designed to combat the consequences of hemolysis in SCD include the use of endogenous plasma haptoglobin (Hp) (protein scavenger) and hemopexin (heme scavenger) to oxidatively inactivate free Hb and heme (34). Hp binding to Hb dimers lowers the redox potential of Hb, stabilizes ferryl heme, and localizes protein radicals on the penultimate Hb βTyr-145, and completely inhibits hemin dissociation until uptake by macrophages (35–37).

The pseudoperoxidative catalytic cycle begins with the ferryl heme formation when ferrous heme reacts with H_2_O_2_. When H_2_O_2_ reacts with the ferric form of Hb, both a protein radical and a ferryl species are formed. The radical is stabilized on the porphyrin or nearby amino acids, which corresponds to the peroxidase compound II heme state. This “unharnessed” radical escapes from the porphyrin ring to other amino acid side chains, including βCys-93, which then reacts with O_2_ to form cysteic acid (25). Both the ferryl heme, with its high-midpoint redox potential (*E*°_1/2_ ∼1.0 V), and its associated protein cation radical induce oxidative changes that affect the Hb protein itself and other biological molecules, which are in close proximity (38). These internal reactions appear to be more exaggerated in HbS and result in the modification of heme, its subsequent attachment to nearby amino acids, and the irreversible oxidation of reactive amino acids, particularly βCys-93, which when oxidized promotes dissociation of hemoglobin into unstable dimers (6).

Because ferryl radicals are known to target βCys-93, we tested whether we could mitigate this damaging process by enhancing reduction and more rapid recycling of the ferryl heme to prevent oxidative damage of this critical amino acid side chain. We first tried to provide a direct electron path in the β subunits to the heme iron by introducing Tyr for Phe at the 41 position and then to add additional electrostatic resistance to oxidation of βCys-93 by introducing Asp at the β 82 position (14).

The reaction of HbS with H_2_O_2_, described in this work, even at stoichiometric levels, occurs much more rapidly and is more damaging to tissues than the simple autoxidation reaction, which produces it (24). Multicomponent calculations of the levels of ferrous/ferric/ferryl intermediates in our spectral measurements showed that the amount of ferryl intermediate in the HbS mutants containing a Tyr at the βC7 helical position (F41Y) and (F41Y/K82D) is significantly less than that seen in reaction mixtures of native HbS or rHbS βK82D, even though the K82D mutant oxidizes at a slightly higher initial rate.

The most striking result is shown in Fig. 6*B* where the amount transient ferryl heme that reacts with Na_2_S to form sulfheme was found to be considerably reduced in rHbS βF41Y and βF41Y/K82D. It has been shown that ferryl iron reduction by a pathway involving Tyr-41 can be enhanced by addition of reductants such as ascorbate (15). Again, our data confirm that ascorbate reduction of the ferryl to ferric iron is enhanced significantly in the βTyr-41-containing proteins.

Our hope was that by reducing the ferryl levels and by shortening its persistence, we could reduce the extent of oxidation of key amino acids. The MS data in Table 3 do show a modest reduction in βCys-93 oxidation in the rHbS βF41Y mutant compared with rHbS, and an even greater decrease in irreversible oxidation of βCys-93 in the rHbS βF41Y/K82D. The data in Table 3 also show that other cysteine residues (βCys-112 and αCys-104) subjected to the same levels of H_2_O_2_ show considerably less oxidation, particularly in HbA. However, it is notable that in all the HbS variants, there were higher levels of β Cys-112 oxidation (∼20% at 2.5 H_2_O_2_/heme) than in HbA (∼ 3%), and this level of oxidation was not affected by the β F41Y or K82D mutations.

To investigate the stabilizing effects of the βK82D mutation on HbS, we carried out a series of experiments where the oxidative and thermal stabilities of the mutant proteins were tested. Incorporating βAsp-82 in HbS together with βTyr-41 provided a small improvement in resistance to thermal denaturation (Fig. 8*B*). However, the more dramatic effect of the βK82D substitution was an increase in the delay times in polymerization and fiber formation for HbS, a much sought-after property in the design of anti-sickling drugs (39). The increased delay time for polymer formation for the rHbS βF41Y/K82D is very pronounced and favorable with respect to preventing sickle-cell disease (Fig. 8*A*).

The question remains how the βPhe-41 → Tyr and Lys-82 → Asp mutations singularly or in combination impact HbS's resistance to oxidative stress, denaturation, and polymerization. Crystal structures of the deoxy and CO forms of HbS have been reported (PDB codes 2HBS and 5E6E, respectively) (40, 41). Both an examination of these structures and recent MS analysis suggest that the relative surface accessibilities for βCys-93 and βCys-112 in HbS are very similar to those for the same amino acid side chains in the deoxy and liganded states of HbA (42). It has been suggested that βGlu-6 → Val at the A-helix may affect the dynamics of the F-helix and the side chains of βCys-93 and Tyr-145 (6). The tyrosine side chain has been suggested to stabilize ferryl globin radicals by oxidation of its ring and is solvent-accessible in the R state (15). Oxidation of βCys-93 will clearly perturb the extensive network of hydrogen bonding and salt bridges at the interface between the β2 FG corner and α1 C-helix, where αTyr-42 is located (29, 41).

Introducing Tyr-41 in the β subunits ∼10.1 Å from the heme iron is in theory capable of facilitating rapid electron transfer (a rate in excess of 1 × 10^8^ s^−1^) (17, 26), depending on the population of both protonated oxoferryl and a deprotonated tyrosine in solutions. The native βLys-82 or mutant Asp-82, in contrast, is located ∼18.3 Å away from the β-heme and is not known to be engaged in electron transfer processes.

We showed previously that the crystal structure of the CO form of Hb Providence, rHb0.1Prov (cross-linked with a glycine residue between the C and N termini of α subunits) is very similar to native HbA-CO (14). The higher oxidative stability caused by the K82D mutation has been attributed to changes in reactivity of the βCys-93 side chain, which may be due to either indirect electrostatic effects or alterations in the dynamics of the structure rather than any large changes in the conformation states of the Hb tetramer (14).

Support for the role of the βK82D mutation in providing structural stability comes from an earlier study, by Weickert *et al.* (43). The recombinant HbProv variant (βK82D) was shown to significantly improve soluble globin accumulation during expression in *Escherichia coli* over its WT recombinant Hb counterpart. It was initially assumed that the βK82D replacement enhanced overall production by making the apoprotein more stable for efficient expression, but our recent work showed that the apoprotein stability of rHb Providence appeared to be similar to that of rHbA (43). Thus, HbProv resistance to oxidative degradation probably plays a key role in enhancing expression in *E. coli* and during purification (14).

The HbProv mutation was reported in the HbS gene of a healthy 30-year-old West African woman. She had a benign form of sickle-cell disease, indicating a beneficial effect of the βK82D mutation. The presence of the HbS mutation (βE6V) was not a clinical problem for this patient, presumably because both mutations were present in the same Hb tetramer (12). The addition of the βK82D mutation to HbS may have provided resistance to both polymerization and oxidative damage in the patient's RBCs, which is supported by our *in vitro* observations in this work.

Most common therapeutic approaches for treating SCD involve the use of small molecule drugs that interact (directly or indirectly) with HbS and reduce its tendency to polymerize by shifting the allosteric equilibrium toward the R quaternary state. Hydroxyurea, which has been in clinics for some time, is known to increase fetal Hb in cells, which acts to terminate the fiber growth by chain termination at γ chains. This reagent, however, appears to have an additional antioxidant benefit as it has been recently shown to promote *S*-nitrosylation of βCys-93 through its NO metabolite (9). l-Glutamine was recently approved by the Food and Drug Administration as an antioxidant, and it works by increasing NAD^+^ levels in SS RBCs and significantly enhancing the redox potential of the cell as defined by the ratio of NADH to the sum of NADH plus NAD^+^ (44).

Gene therapy, in which permanent delivery of a corrective or an anti-sickling gene cassette into long-term, repopulating autologous hematopoietic stem cells, can potentially produce permanently corrected RBCs in patients and is another active area of therapeutic development. However, donor availability and immunological complications are some of the hurdles facing this approach. Another gene therapy approach includes the use of lentiviral gene and genome editing. Lentiviral methods have advanced considerably and have led to the development of anti-sickling globin vectors carrying human β-globin to correct polymerization of HbS. Synthetic β-globin variants, with site-specific mutations affecting axial and lateral contacts in the sickle-cell fiber by a single mutation, βT87Q, are currently under clinical evaluation (45). A triple combination of amino acids (βG16D/βE22A/βT87E) known as HbAS3 has also been developed (46).

Ho and co-workers (29) have also examined mutations in α chains of HbS, which improve solubility and dramatically increase delay times. In combination, His → Gln mutations in α subunits at the axial (position 20) and lateral (position 50) contacts in the HbS fiber cause marked 3-fold increases in solubility and dramatic increases in delay times that are even larger than that shown for the β^S^ F41Y/K82D mutant in Fig. 8. However, in unpublished work^4^ with these variants, we observed little effect of these α substitutions on the resistance of the HbS variant to oxidation and radical formation. If the effects were additive, it would be interesting to consider the favorable properties of an HbS variant with α(H20Q/H50Q) and β^S^(F41Y/K82D) substitutions. Such an HbS is likely to be as soluble and resistant to oxidation as HbA.

Our work shows that introduction of βTyr-41 in HbS reduces ferryl heme content in the presence of H_2_O_2_ leading to a significant improvement in oxidative stability and that inhibition of HbS gelation can be achieved by introduction of βAsp-82. In combination, these mutations lead to a dramatic increase in the resistance of βCys-93 to oxidation and in the gelation time of HbS. These favorable effects occur despite the fact that both amino acids, when introduced singularly or in combination, lead to a “right” instead of a “left” shift in oxygen-binding curves. In the past, decreases in *P*_50_ were thought to be an essential requirement for the inhibition HbS polymerization. Finally, our data suggest that gene-based intervention strategies should also consider mutations that affect the oxidative pathways for HbS degradation in addition to the oxygen-binding properties of HbS, when considering globin engineering strategies.

## Experimental procedures

### Proteins and chemicals

Human Hbs (A and S) were purified as described previously using anion and cation chromatographies (23). The complete removal of antioxidative enzymes, namely superoxide dismutase and catalase in the purified Hb solutions, was verified as reported previously (47). All chemicals and reagents were purchased from Sigma or Fisher unless otherwise indicated. Gases were purchased from Roberts Oxygen Co., Inc. (Rockville, MD). H_2_O_2_ (30% w/w) was purchased from Sigma. Dilute solutions of H_2_O_2_ were prepared fresh daily from a stock solution by making appropriate dilution in deionized water and kept on ice. The concentration of H_2_O_2_ was determined spectrophotometrically at 240 nm using a molar extinction coefficient of 43.6 m^−1^ cm^−1^ (48).

### Recombinant protein expression and purification

The HbS WT and mutant recombinant proteins were expressed using the pHE2 plasmid (49, 50). Briefly, α-globin and β-globin genes are in tandem on the plasmid and co-expressed with methionine aminopeptidase under a *tac* promoter, resulting in authentic hemoglobin without an extra N-terminal methionine. All amino acid substitutions were made using Stratagene PCR-based “QuikChange^TM^ site-directed mutagenesis” kit (Stratagene, La Jolla, CA). The *E. coli* strain JM109 was used for mutagenesis and expression of hemoglobin using the pHE2 system.

Large-scale Hb expression was done in a Biostat C20 bioreactor (B Braun Biotech International, Melsungen, Germany) following procedures very similar to those described by Looker *et al.* (51), Shen *et al.* (50), and Strader *et al.* (14). Cells were grown in DM media, initially at 37 °C, pH 7.0. Steering speed and airflow were controlled automatically to keep the oxygen level at ∼60%. The glucose (40% w/v solution) feeding profile was the following: 40 ml/h for 0–0.5 *A*_600_; 60 ml/h for 0.5–1 *A*_600_; 100 ml/h for 1–1.6 *A*_600_. After the *A*_600_ reached 1.6, the temperature was decreased to 28 °C, and protein expression was induced by isopropyl 1-thio-β-d-galactopyranoside (up to final concentration 0.05 g/liter). Excess heme was added throughout the expression phase at a feeding rate of ∼100 ml/h (1 g/liter solution), and glucose (40% w/v solution) was supplied at ∼50 ml/h. The expression phase was maintained for 9–12 h, and then the cells were harvested. The final suspension was bubbled with 1 atm of CO, pelleted by centrifugation, and frozen for storage.

For protein purification, cells were thawed, purged with CO, and resuspended in lysis buffer (40 mm Tris, pH 8.5, 1 mm EDTA, 20 mg of DNase, 134 mg of benzamidine, 100 mg of lysozyme) until a uniform suspension was obtained. The slurry was then filtered through cheesecloth and run through a cell breaker (Avestin, Inc., Canada) twice. Protein isolation and purification were performed following the basic outline (50, 51). After addition of 2–5 ml of SUPERFLOC C-573, an anti-coagulating agent, the slurry was centrifuged to remove precipitates. Zinc acetate (2 mm) was added to cell lysate; the pH was adjusted to 8; and sample was loaded on a zinc IMAC column. The column was washed by sequential passage of the following: (*a*) 20 mm Tris/HCl, 500 mm NaCl, pH 8.5 (3 column volumes (CV)); (*b*) 200 mm Tris/HCl, pH 8.5 (2 CV); (*c*) 20 mm Tris/HCl, pH 8.5 (3 CV). Hb was eluted with 20 mm Tris/HCl, 15 mm EDTA, pH 8.5, buffer. The eluent was ∼85% recombinant CO-Hb (rHbCO) and purified further using anion- (Sepharose Q) and cation-exchange (Sepharose S) chromatography. All procedures were carried out at 4 °C, using ice-cold CO-purged buffers, and the protein was always kept under a CO atmosphere. Sample purity was judged by reverse-phase HPLC of the denatured α and β polypeptide chains, isoelectric-focusing gel electrophoresis of the HbCO forms, and accurate molecular mass measurements using the University of Wisconsin-Madison Biotechnology Center MS/Proteomics Facility.

### Spectrophotometry and hemoglobin concentration measurements

Hb concentrations were calculated on a heme basis. The levels of ferrous/oxy-Hb, ferric/metHb, and ferryl Hb were measured based on the absorbances at λ = 541, 576, 630, and 700 nm using recently published extinction coefficients (23). Spectrophotometric measurements were carried out in a UV-visible diode array spectrophotometer (Agilent HP 8453).

### Autoxidation reactions of hemoglobins

Autoxidation experiments were carried out by incubating Hb samples in 50 mm phosphate buffer, pH 7.4, at 37 °C for 24 h. Absorbance changes in the range of 350–700 nm due to the spontaneous oxidation of oxy-Hbs (60 μm in heme) were recorded in a temperature-controlled photodiode array spectrophotometer (HP 8453). Multicomponent analysis was used to calculate the oxy-Hb and metHb concentrations based on extinction coefficients of each species (23). Time courses for autoxidation were represented in logarithmic plots of −ln([oxy-Hb]*_t_*/[oxy-Hb]_0_) *versus* time *t* (hour) (52).

### Hydrogen peroxide-mediated oxidation and ferryl hemoglobin formation

The spectra of reaction mixtures of ferrous Hbs (60 μm) with molar excess H_2_O_2_ (60, 150, and 600 μm) were monitored every 20 s for 30 min in a photodiode array spectrophotometer (HP 8453). After completion of the reaction, 2 units of catalase were added into the reaction mixture (1 ml) and incubated for 1 min to remove excess H_2_O_2_. Ferryl Hb formation was verified by adding 2 mm sodium sulfide (Na_2_S), which transforms the ferryl Hb to a sulfHb (6, 53). The levels of ferryl Hb were estimated using an extinction coefficient at 620 nm for sulfhemoglobin. Ferryl Hb (1 ml) was also generated by reaction of ferric Hb (60 μm) with excess H_2_O_2_ (600 μm) in PBS for 90 s. Six units of catalase were added into the reaction mixture to remove the excess H_2_O_2_.

### Stopped-flow kinetics of the reduction of ferryl hemoglobin by ascorbate

Time courses for ferryl reduction (5 μm) to the ferric state by ascorbate (0–1000 μm) were monitored using an Applied Photophysics SX20 stopped-flow spectrophotometer in PBS at room temperature as reported previously (6, 54). Spectra were captured as a function of time using an Applied Photophysics photodiode array accessory, and the spectra at various time points were analyzed using multiple wavelengths. For each reaction, at least three kinetic traces were averaged and fit to exponential equations using the Marquardt-Levenberg fitting routines included in the Applied Photophysics software.

### Kinetics of heme loss from ferric hemoglobins

Absorbance changes were monitored after mixing the double-mutant apomyoglobin (apoMb) (heme acceptor) (H64Y/V86F) with samples of metHbS. The apoMb reagent binds heme released from ferric Hb generating a spectral unique “green” holoMb end product as described previously (22). Absorbance spectra between 350 and 700 nm were recorded every 2 min for 16 h at 37 °C using 200 mm potassium phosphate buffer at pH 7.0 to which 600 mm sucrose was added to prevent denaturation of the resultant apoHb. To carry out this experiment under first-order reaction kinetics, excess heme-binding ApoMb was used. The final concentration of ferric Hb in heme equivalents was 2 μm, and the final concentration of H64Y/V86F apoMb was 20 μm in a 1-ml reaction volume. Data collection started immediately after mixing the two solutions; absorbance changes at 410 nm were used for the calculation of the extent of heme transfer.

### Measurements of hemoglobin polymerization kinetics and delay times

The delay time for HbS polymerization is defined as the time of deoxygenation of the sample and the appearance of polymers (29, 55). To determine the effects of mutations on the delay time, polymerization progress curves were obtained using the temperature-jump technique described previously by Adachi and Asakura. (27). Anaerobic cuvettes (with a 1-cm path length) sealed with rubber caps were used for the photometric measurements. The reaction was carried out in 1 ml of phosphate buffer (1.8 m, pH 7.3), and sodium dithionite (Na_2_S_2_O_4_) (0.05 mg) was added to the cuvette and sealed. Two needles were inserted into the rubber cap for gas inlet and outlet. Nitrogen gas was then bubbled through the needle into the solution for 30 min with gentle pressure. The temperature of the cuvette was brought to 0 °C immediately. A stock Hb solution was then aliquoted (at 0 °C) in the cuvettes through a rubber septum with a Hamilton gas-tight syringe to make a final concentration of Hb in the cuvette of 100 μm. The cuvette was then placed in a preheated sample holder in the spectrophotometer (30 °C). The change in turbidity was measured with a temperature-controlled photodiode array spectrophotometer (Agilent 8453, Santa Clara, CA) at 700 nm. Absorbance at 700 nm (on the *y* axis) was plotted against time (on the *x* axis abscissa). The intercept of the slope with the abscissa was determined as the polymerization delay time (56).

### Thermal stability of hemoglobins

Thermal stability measurements were performed using a method described previously (29). OxyHb (50 μl, 2 mm/heme) solutions in 100 mm potassium phosphate, pH 7.4, were used. Samples were inserted into a PCR that was programmed to run at 60 °C for 10 min and then cooled to 25 °C in 1 min. The precipitates in the samples were removed by centrifugation (1000 rpm for 5 min), and the absorbance of the supernatants at 577 and 700 nm was recorded after appropriate dilution. The readings at 700 nm were treated as background and subtracted from the 577 nm readings. Samples without heating step were used as controls, and the amount of precipitated proteins was calculated accordingly and expressed as a percentage.

### Oxygen equilibrium studies

OECs for the Hb solutions were obtained using a Hemox analyzer (TCS Scientific, New Hope, PA). Oxygen tension was measured with a Clark-type oxygen electrode, and Hb saturation was measured using a built-in dual-wavelength spectrophotometer. The concentration of Hb samples was between 60 and 75 μm (heme), and the temperature was maintained at 37 °C. To maintain the metHb content to a minimum level, 4 μl of the Hayashi enzymatic reduction system was added to the final solution (3 ml) (57). A computer-based analysis of oxygen-binding curves was performed yielding *P*_50_ (partial pressure of oxygen at which Hb is half-saturated) and *n*_50_ (Hill coefficient) for oxygen binding. Oxygen equilibrium parameters were derived by fitting the Adair equations to each oxygen equilibrium-binding curve by the nonlinear least-squares procedure included in the Hemox analyzer software (p50 PLUS, Version 2.0).

### Isoelectric focusing analyses

IEF analysis of Hb and variant samples was carried out on precast IEF-agarose gels (Invitrogen®, pH 3–10, IEF gel). The gels were electrofocused using a Novex® power supply at 100 V for 1 h, 200 V for 1 h, and 500 V for 30 min. The IEF gels were fixed in 12% TCA for 30 min.

### Reversed-phase high-performance liquid chromatograph (RP-HPLC)

RP-HPLC was performed with a Zorbax 300 SB C3 column (4.6 × 250 mm) coupled to a Waters HPLC system consisting of a Waters 626 pumps, 2487 dual-wavelength detector, and a 600-s controller installed with Empower 2 (Waters Corp.) (6). 20 μg of Hb in 25 μl of water was loaded onto a C3 column equilibrated with 35% acetonitrile containing 0.1% TFA. Globin chains were eluted with a gradient of 35–50% acetonitrile within 100 min at a flow rate of 1 ml/min. The eluent was monitored at 280 nm for globin chains and at 405 nm for the heme components.

### MS analysis of recombinant hemoglobins reactions with peroxide

Quantitative mass spectrometry (MS) analysis was performed utilizing 60 μm (heme) native HbA, recombinant HbS (rHbS), the rHbS mutants βF41Y, βK82D, and βF41Y/K82D respectively. To study the effect of increasing H_2_O_2_ concentrations on Hb hot-spot amino acid oxidation, the following experimental conditions were utilized for all MS experiments described below. Experiment 1: controls, Hbs were incubated in air-equilibrated PBS buffer without H_2_O_2_. Experiments 2 and 3: Hbs were incubated with 2.5 and 10 molar excess of H_2_O_2_ per heme. All oxidation reactions were carried out in PBS, pH 7.4, at ambient temperature for 30 min. 1 μl of 1 unit/μl catalase was added to remove excess H_2_O_2_ to quench oxidation from samples listed in experiments 2 and 3.

### LC-MS/MS analysis

All Hb samples (listed above in experiments 1–3) were digested with trypsin, desalted, and analyzed by MS using our previously described method (58). Briefly, tryptic peptides were analyzed by reverse-phase LC/MS (RP LC/MS/MS) using an Easy nLC II Proxeon nanoflow HPLC system coupled on-line to a Q-Exactive Orbitrap mass spectrometer (ThermoFisher Scientific). Data were acquired using a top10 method (for 60 min), dynamically choosing the most abundant precursors (scanned at 400–2000 *m*/*z*) from the survey scans for HCD fragmentation.

Mass spectrometry was also used to confirm relevant mutations for each recombinant Hb and demonstrate that each sample was catalase-free by searching data against the Swiss-Prot Human database (release 2014_03, containing 542,782 sequence entries) supplemented with β sequences containing E6V, E6V/F41Y, and E6V/K82D mutations and porcine trypsin using the Mascot (Version 2.4) search engine (Matrix Sciences, London, UK) as described previously (58). Variable modifications, including cysteine trioxidation (+48 Da), tryptophan oxidation (+16), tyrosine oxidation (+16 Da), and methionine oxidation (+16) were included for identifying hot-spot oxidations (25, 59). Mascot output files were analyzed using the software Scaffold 4.2.0 (Proteome Software Inc.). Hb peptide identifications were accepted if they could be established at greater than 99.9% probability and contained at least two identified peptides. Probabilities were assigned by the Protein Prophet algorithm (60).

### Quantitative MS analysis

Peptides from LC-MS/MS data were analyzed to quantify changes in rHbs under the oxidative conditions used in the rHb experiments as described previously (58). Each peptide was further validated by retention time reproducibility. All quantitative experiments were performed in triplicate, and standard deviations were obtained by averaging relative abundance data from three different experiments. Extracted ion chromatograms (XICs) were generated from the most abundant monoisotopic peak of isotopic profiles representing charged states of each peptide. To construct XICs, Xcalibur (Version 2.2) software was used with a designated mass tolerance of 0.01 Da, and mass precision was set to three decimals. For relative quantification, the ratio of each isoform was calculated based on the sum of the XIC peak area from all forms, which was normalized to 100% and included all charge states and versions that result from different cleavage sites.

## Author contributions

F. M., T. K., and M. B. S. formal analysis; F. M., T. K., and M. B. S. investigation; F. M., T. K., M. B. S., J. S., and J. S. O. methodology; F. M., T. K., M. B. S., J. S., and A. I. A. writing-original draft; J. S. O. and A. I. A. validation; J. S. O. and A. I. A. writing-review and editing; A. I. A. conceptualization; A. I. A. supervision; A. I. A. project administration.

## Footnotes

4 F. Meng, T. Kassa, M. B. Strader, C. Ho, and A. I. Alayash, unpublished data.

## Footnotes

3 The abbreviations used are:

SCD: sickle-cell disease
IEF: isoelectric focusing
E6V: hemoglobin with βGlu-6 → Val mutation
HbA: human adult hemoglobin
HbS: sickle-cell hemoglobin, HbA with βGlu-6 → Val (E6V) mutation
Hp: haptoglobin
Mb: myoglobin
MP: microparticle
OEC: oxygen equilibrium curve
RBC: red blood cell
rHbS: recombinant hemoglobin with βGlu-6 → Val mutation, wildtype HbS
rHbS βF41Y: recombinant sickle-cell hemoglobin with additional βPhe-41 → Tyr mutation
rHbS βF41Y/K82D: recombinant sickle-cell hemoglobin with additional βPhe-41 → Tyr and βLys-82 → Asp mutations
rHbS βK82D: recombinant sickle-cell hemoglobin with additional βLys-82 → Asp mutation
RP-HPLC: reversed-phase HPLC
PDB: Protein Data Bank
CV: column volume
ROS: reactive oxygen species
HbProv: Hb Providence
XIC: extracted ion chromatogram
rHb: recombinant Hb.
